# When Is Social Value Proportionate to Research Risks?

**DOI:** 10.1111/bioe.70109

**Published:** 2026-06-01

**Authors:** Robert Steel

**Affiliations:** ^1^ Clinical Center Department of Bioethics US National Institutes of Health Bethesda Maryland USA

**Keywords:** proportionality, research ethics, risk‐benefit assessment, risk‐benefit ratio, social value

## Abstract

Ethical human subjects research must have an acceptable risk‐benefit ratio, which requires that the net risks participants face be proportionate to the research's social value. Yet existing scholarship does not explain what makes risks proportionate to social value. Here, I distinguish internal and external senses of proportionality and argue that it is internal proportionality that is the proper object of IRB/REC review. I propose understanding internal proportionality in terms of a literal ratio of expected health effects; I also argue against the alternative of attempting to assess proportionality through intuitive comparisons. I illustrate the potential implications of this account through a discussion of the proposal to engage in controlled human infection studies with the novel coronavirus. This case highlights that trials with enormous numbers of potential beneficiaries can be internally proportional even when they are very unlikely to succeed.

## 1 Introduction

It is widely agreed that, in order for research to be ethical, the risks it imposes on its participants must be justified by proportionate social value.^1^ However, there is no existing account of what proportionality between risk and social value consists in, and, relatedly, no accepted method for determining when it obtains. This paper demonstrates this gap, argues for a particular way of closing it, and sketches implications.

The project is motivated by recent debates over the permissibility of controlled human infection trials (CHIs) using the novel coronavirus, that is, trials which would intentionally expose healthy volunteers to SARS‐CoV‐2. Early in the pandemic, some proposed such CHIs as a method for accelerating vaccine development [3, 4], leading to a robust scholarly discussion [5, 6, 7]. While early proposals were criticized on a number of grounds, the most compelling among them was that CHIs were, for various logistical reasons, very unlikely to succeed in accelerating vaccine licensure, and that they therefore lacked proportionate social value.^2^ In other words, the claim was that even if such CHIs' risks to participants were low enough to be in‐principle justifiable, their actual value was insufficient to do that justifying. Unfortunately, the undertheorized nature of the proportionality relationship left it unclear how to determine whether the claim of insufficient value was true.

This paper attempts to advance our understanding of proportionality. It distinguishes two different kinds of proportionality, internal and external, and argues that it is internal proportionality that should be the primary object of research ethics review. It proposes understanding internal proportionality as a requirement that at least a certain fixed numeric ratio hold between a study's risks and benefits and argues that, so understood, the most plausibly required ratio is “1 or less.” It illustrates how, due to the vast imbalance between the numbers of people in a trial and those who stand to benefit, scientifically rigorous trials addressing widespread health conditions have a strong tendency to meet this standard, and that his can be true even when they are very unlikely to succeed. This account is defended from objections and alternatives are criticized.

The arguments to follow are preliminary in character. Investigating an under‐discussed topic requires judgment regarding what to argue and what to presuppose, and it is often not possible to address all possible positions. If some of these judgments were mistaken, perhaps this will at least prompt future work correcting them. That would remain a salutary result, as the nature of the proportionality requirement could only benefit from further study.

## 2 Distinguishing Proportionality From Other Requirements

According to the most prominent framework, there are seven distinct ethical requirements on clinical research: social value, scientific validity, fair subject selection, favorable risk‐benefit ratio, independent review, informed consent, and respect for enrolled subjects [10]. Most of these are orthogonal to proportionality; it is possible for a study to obtain the informed consent of its participants even if its risks are disproportionate to its benefits, and similarly possible for a study to have risks which are proportionate to benefits but nonetheless to fail to obtain participants' informed consent. Proportionality enters the picture specifically as a sub‐component of the requirement for a favorable risk‐benefit ratio.

Here, I follow Rid and Wendler's seminal exposition of that requirement [11].^3^ First, a favorable risk‐benefit ratio requires that risks be minimized and benefits maximized. Second, it requires that the “net risks” of the research to individual participants—that is, the degree to which the risk to the participant of participation exceeds the potential benefits—that remain after that risk minimization and benefit maximization must not be too high in absolute terms (this forbids, e.g., offering even competent adults the opportunity to enrol in a trial which would predictably kill them, regardless of how socially valuable the potential resulting knowledge might be). And third, it requires that those not‐too‐high net risks must then be justified by proportionately large potential social value.

As is commonly noted, the standard language of comparing “risk” and “benefit” is unfortunate as these terms are not parallel; unlike the idea of risk, the idea of benefit does not include qualification by chance. While I use the standard language, the benefits I compare against risks should be understood throughout as benefits as assessed in expectation and qualified by chance, like risks, rather than as benefits *simpliciter*. Similarly, I assume that risks and benefits to individuals can ethically cancel each other out, and similarly for risks and benefits to society, and hence that the interesting normative question concerns how to relate the margins of each that remain after such cancelling. That is, the question of proportionality regards the sort of justificatory relationship that must hold between net risks to participants and net benefits to society. Reflecting this, and for terminological simplicity, in the remainder I replace talk of research's “social value” with talk of its “benefits,” and both here and throughout, “risks” and “benefits” should be understood as referring to *net* risks and benefits.^4^

Because this paper seeks to discuss proportionality specifically, it assumes for the sake of argument that all other standard ethical requirements mentioned here are both genuine requirements and satisfied in all cases under discussion. How should we understand a *further* requirement that risks must be justified by proportionate benefits, and how can we know whether this further requirement is met or not? For instance, what does it look like for a socially valuable, scientifically valid… and so forth. study to have risks that are minimized, benefits that are maximized, and risks that are not too high in absolute terms, yet nonetheless be unethical to conduct *just by virtue of* those (minimized, not‐too‐high) risks failing to be justified by proportionate benefits?

## 3 Existing Tests for Proportionality

The most developed existing test for proportionality is Rid and Wendler's ideal social arbiter test. It goes as follows: imagine that the risks and benefits of a trial were evaluated by an arbiter that was fully informed and impartial, gave all claims fair consideration, and treated like cases alike; if such an ideal arbiter would endorse a study, then its benefits justify its risks, whereas if it would oppose or be indifferent to it, then they do not [11, pp. 165–167].

This test successfully rules out some obviously unacceptable ways of assessing proportionality, for example, relying on partial or unfair considerations. It also requires consistency across different arenas of social policy, which enables helpful comparisons. But beyond those constraints, what such an arbiter's concerns would be and its method of assessing them are unspecified. While the arbiter is ideal and hence will presumably make the correct determination, we lack a method to tell us what that determination is.

The Net Risks view is not alone in lacking a substantive answer regarding what proportionality consists in; no other account describes this, and the ideal arbiter device offers at least some guidance. Nonetheless, even in the absence of an available explanatory theory, it may be that practitioners still share an implicit understanding. A charitable interpretation of the ideal arbiter test is that it is not intended as a substantive normative explanation, but instead as a helpful imaginative device for practitioners to use in honing their intuitive judgments.

One won't be able to detect such a shared understanding just by looking to the frequently deployed language of reasonability and justification: the claims that risks to participants must be “justified by,” or “reasonable in relation to” a trial's prospective social value seem only to say that, whatever relationship between the two matters, it should hold. But there is also thicker language that builds in more structure: the language of *weighing* risks against benefits and of a favorable risk‐benefit *ratio*. Weighing metaphors suggest that what is being compared is two magnitudes, the term “favorable ratio” suggests that the number representing the magnitude of the risks, divided by the one representing the magnitude of the benefits, must be sufficiently small. In what follows, I develop the proposal that risk‐benefit ratios are literally ratios.

## 4 Internal Versus External Proportionality

What sort of (literal) ratio would represent a proportionate relationship between risks and benefits? One answer: there is some absolute numeric threshold—for instance, a requiring a ratio of 0.25 or less would require that risks always be justified four‐times‐over by benefits. Call this understanding “internal proportionality.” A different answer: there is no single threshold, but instead acceptability variably depends on background facts. Most plausibly, these would include facts about other studies: benefits four‐times‐over may sound good in the abstract but nonetheless be inadequate if put forward against a backdrop of other studies offering benefits eight‐times‐over. Perhaps studies must be as good, or nearly as good, as the best in some relevant comparison class, which might include all other research, or a more limited class of other research sharing certain specified features (perhaps: socio‐behavioral, healthy volunteer, phase 1, etc.) Call this understanding “external proportionality.”

For both kinds of proportionality, it is important to remember that, as above, an evaluation of proportionality occurs *after* risks have been minimized and benefits maximized, for example, redundant burdensome research procedures have already been omitted, the quality and relevance of data already maximized, and so on. Proposed studies must already be, in some sense, the best version of themselves before they are ready for proportionality tests. The question of internal proportionality is then how to assess the balance of risks and benefits within that optimized version, and the question of external proportionality is how the balance within that optimized version compares to the balance of other also‐optimized actual or potential studies in the appropriate class.

With this distinction and clarification in hand, ask: which of these notions of proportionality, internal or external, ought to concern us? In fact, both internal and external proportionality are worthy ideals. We have reasons to want trials to be good enough, as considered on their own; we also have reasons to hope that trials which more efficiently convert risk into benefit are conducted rather than those that do so less efficiently. So rather than picking only one standard as normatively relevant, which should be assessed depends on the context. We should think about who is doing the assessing and what the effects of their using different standards would be.

Here, I will focus on the decision by Institutional Review Boards (IRBs) and other Research Ethics Committees (RECs) to either approve or reject proposed research studies. US Federal regulations require IRBs to assess studies risk‐benefit ratio, and these decisions affect what kind of research can go forward. This correspondingly shapes what kind of research is conceptualized and proposed, as researchers and funders have no incentive to develop studies that cannot pass review. Given that the standards for independent review shape the research landscape in this way, it is worth investigating how these bodies should understand proportionality. Here, I argue that RECs may reasonably assess internal proportionality but ought not assess external proportionality. This is because while improving the external proportionality of research is a worthwhile goal, it is doubtful that rejecting individual studies under REC review is a morally appropriate or practically feasible way of advancing it.

If RECs were to only approve research with favorable external proportionality—studies whose risk benefit ratio is at least approximately close to the best on offer in the relevant comparison class—this would effectively forbid research stakeholders, for example, participants, researchers, and funders, from carrying out inefficient research. But this would conflict with the common belief that agents have moral prerogatives that include the option to sometimes permissibly do less than what is overall best, including when doing so expresses or advances their personal values, interests, and commitments. It may be that between two healthy volunteer studies with equivalent risks, a participant could achieve a better return on risk by enrolling in the study related to heart disease than the one related to dementia, yet that participant may wish to honor a family connection to dementia. It may be that a particular researcher could also achieve a better return by launching a study investigating a common variant of a disease rather than a rare one, yet they may be intellectually passionate about the rare variant. A foundation which raised money via disease pledges may be not only be permitted, but in fact obligated to fund research into those diseases rather than others, even in cases where that other research would have a better overall balance of risk and benefit [14, p. 7]. If, as commonly assumed, it is morally unproblematic for agents to have and act on preferences which do not strictly track what is impersonally best, it is unclear why it would be appropriate for RECs to forbid them from doing so in this specific context. This is especially so when we remember that many of those stakeholders have no prior obligation to participate in or advance research at all, leaving it puzzling why they should be barred from supporting research which, while perhaps worse than the best, is still worthwhile when considered in itself.

This case for RECs *not* preventing trials with poor external proportionality from going forward depends on a putative liberty to help others in marginal ways. Perhaps one is not impressed and instead thinks that regulatory authority should simply be used to do the most good, and one also thinks that the most good would be done by RECs rejecting trials with poor external proportionality. Importantly, this claim is questionable for practical reasons independent of the above argument. RECs are in a poor position to assess external proportionality, as it requires not just knowledge of an individual trial, but also of other, competing opportunities for research. But an REC is asked to review a particular protocol and may have limited ability to compare it to competing or hypothetical studies. Even if an REC is able to produce a sound assessment, it is limited in the degree to which it can appropriately follow through. A finding of poor external proportionality would, ideally, result not only in steering research resources away from the lower‐value trial, but also in ensuring that it actually goes to a higher‐value one instead. But RECs lack the authority to redirect resources: they can reject a protocol, but cannot order the conduct of another, better one in its place. Rejecting internally proportionate studies at the point of REC review thus risks letting the perfect be the enemy of the good.

A number of stakeholders other than RECs can play a role in promoting the conduct of externally proportionate research, including researchers themselves and even participants, provided they are appropriately informed. However, the best‐positioned stakeholder is likely large funders: they have access to information not only about one proposed trial, but about a range of trials being conducted within and across research areas; they also have the resources and expertise to generate, synthesize, and evaluate relevant information to make necessary comparisons. Furthermore, they are able to not only decline to fund studies with poor external proportionality, but to direct those very same funds to the support of a different, better studies. And given that they are choosing where to allocate their own resources, rather than placing restrictions on others, they do not inappropriately constrain anyone else's liberties when they do so.

For the foregoing reasons, external proportionality is more appropriately and effectively addressed by stakeholders other than RECs, at least as they are currently resourced and empowered. By contrast, it is significantly more reasonable for RECs to assess internal proportionality. While these assessments are also extremely difficult, RECs at least have access to and are anyway already assessing the protocol under submission. Furthermore, their ability to reject studies is appropriately applied to studies which lack internal proportionality: if such studies fall underneath an absolute threshold below which they should not be conducted, preventing them from being conducted is an appropriate remedy.

## 5 Why the Appropriate Internal Ratio Is “1 or Less”

But what makes a study internally proportionate? If risk‐benefit ratios are literally ratios that are being compared against an absolute threshold, then we can explain internal proportionality by specifying what makes up the magnitude of risk, what makes up the magnitude of benefit, and what fixed value the ratio between them must attain.

Different choices are possible.^5^ For expository purposes, I make three simple assumptions: (1) the magnitudes being assigned are health‐related; (2) that group magnitudes should be arrived at by summation from individual ones; and (3) that prospective uncertainty in health effects should be accounted for through calculating expected values. Given these choices, a trial's risk‐benefit ratio is the expected health risk to participants of running it, divided by the expected health benefits to society running it:

$$\text{Risk}-\text{Benefit}\;\text{Ratio}{=}_{\text{def}}\left(\begin{array}{l} \text{probability}\;*\;\text{magnitude}\;\text{of}\;\text{health}\;\text{risks}\;\text{to}\;\text{each}\;\text{participant},\text{summed} \end{array}\right)\;/\left(\begin{array}{l} \text{probability}\;*\;\text{magnitude}\;\text{of}\;\text{health}\;\text{benefits}\;\text{to}\;\text{each}\;\text{non}-\text{participant},\text{summed} \end{array}\right)$$

In interpreting, remember that, as throughout, these are *net* risks and benefits. Also, notice that the formula assumes that the research poses both net risks to participants and net benefits to non‐participants. This is intentional. There is no need to consider trials with net risks to society, as these should not be run on grounds independent of proportionality; there is also no need to consider trials with net benefit to participants, as the (gross) participant risks are already justified on similarly independent grounds, that is, directly by the (gross and larger) participant benefits. Finally, note also that, insofar as proportionality concerns the justification of net participant risks, it is primarily a concern for studies which solicit informed consent from competent adults, given that research in populations which cannot provide informed consent is sharply limited in the (net) participant risks it is permitted to impose. In the arguments to follow, I therefore assume that any research under discussion solicits informed consent.^6^

So, assuming both risks to consenting participants and social benefit, what value must their ratio attain in order to represent a proportionate and justifying relationship? It seems clear that internal proportionality should require at least that the result of dividing a trial's risks by its benefits be less than one, that is, that the aggregate expected effect on both participants and non‐participants be beneficial. But should the required ratio be substantially less? Plausibly, no. The reasons are similar to the already‐described ones for which IRBs should focus on internal rather than external proportionality, namely, not letting the better be the enemy of the good and consideration of the liberty of stakeholders to pursue marginally helpful projects.

Provided that potential participants are informed, competent, and consenting adults, and provided that the individual net risks are not too high—recall that the individual net risk ceiling is a *separate* requirement from proportionality and not under question here—their enrolling in a proposed trial with a ratio less than one would be a situation in which open‐eyed volunteers offered to shoulder a burden which is not too large in absolute terms in order to produce a greater benefit for others. Restraining them from doing so would apparently be an instance of what Chappell has termed *anti‐beneficent paternalism* [15]. In protecting people from the risks they themselves freely chose in order to provide greater benefits to others, anti‐beneficent paternalism unattractively both insults individual autonomy and makes overall outcomes worse.

This argument appeals to respect for potential participants' autonomous choices. But it is possible to doubt the autonomy of those choices given common participant misconceptions. Here, specifically, one might be skeptical that participants would ever really autonomously seek to enrol in research which was only marginally beneficial.^7^ I am sympathetic to this style of argument and accept that it plays a role in explaining why, despite considerations of participant autonomy, the ratio should not be allowed to be *greater* than 1—few have values and attachments that attract them to trials which are net negative in expectation, or few enough anyway that their accommodation is not a major consideration in social policy.^8^ But when choosing among a set of generally pro‐social options, it is common for individuals to blend consideration of social benefit with their other interests and commitments.

To illustrate, return to the earlier example of SARS‐CoV‐2 CHIs. Early in the pandemic, many were moved by altruistic motivations to sign up as volunteers for such trials [17]. If that motivation consisted solely in a desire to do the greatest quantity of good, and if critics were correct that the benefit associated with those trials was marginal, then interest in participation may have been based on a social value misconception [18]. However, motivation by altruism does not imply a sole concern with doing the most good. Nearly all surveyed volunteers ranked helping others and potentially saving lives as among their top three reasons for wanting to enrol in a COVID CHI (95.9%). Nonetheless, some also included self‐directed reasons such as for example, curiosity about COVID (8.9%), payment (4.1%), and guaranteed access to critical care (8.2%); around one‐fifth included the (inscrutable) “another factor not mentioned” (19.9%) [17], [note 4, table 2].

Consider an analogy to undirected organ donation. Undirected organ donors commonly report motivations which meld other‐directed altruism with consideration of how their own lives go—valuing, for instance, identity‐formation as a donor, the opportunity to give an unreciprocated gift, and wanting to leave a positive legacy [19]. Given that most do not express regret, but instead reported feelings of fulfilment, contentment, and pride, motivation by a desire to donate as part of a positive life‐conception would not apparently be inapt [19]. Furthermore, these desires are consistent with undirected organ donation not being the most efficient way of helping others (e.g., if smaller overall investments of time and resources could do more good when donated to a cost‐effective charity).^9^ An individual may value helping others, but at the same time may want to do so in a particular embodied, socially, and personally significant way, and this is as intelligible as a motivation for the embodied helpfulness of challenge participation as it is for undirected donation.

Of course, these observations notwithstanding, it is important that any potential participants be informed of the best possible estimates of a study's social value—avoiding potential misconceptions is important. And it is expectable that in a study with borderline internal proportionality, adequate disclosure would lead some to lose interest in enrolling. The point is only that we should not assume that no one could or would remain interested after that disclosure, or, that if they did, it could only be because of some uncontroversial failure in understanding or reasoning; respect for autonomy thus remains a relevant consideration.

## 6 Implications of “Less Than 1”

Accepting “less than 1” as the relevant threshold, how can we recognize when it is met? Calculating the ratio for a specific trial would be extremely complex. Factors to consider include, at minimum, the estimated health burden of the target disease and size of health benefit of any potential interventions, size of the affected population, priority of benefiting the affected population, the novelty, innovation, and quality of the research questions being investigated, the rigor of study design and feasibility of conducting it, the quality of reporting and dissemination of results, and influence on future research and public health practice [21]. Such evaluations are not impossible, but they are significant research projects in their own right. Fortunately, even without conducting one here, it is possible to say informative things about the implications of assessing proportionality through literal ratios.

The important points are, first, that requiring a specific ratio does not impose any absolute constraints on the value of any single term: ratios are relational, and it follows that both arbitrarily low chances of success and arbitrarily high participant risks can always be offset, provided that enough counterbalancing weight is added elsewhere. Of course, arbitrarily high participant risks will be independently ruled out by the already‐mentioned standard postulation of an absolute ceiling on acceptable net risks. Low chances of success, however, will not be. And the second important point is that the population that stands to benefit from a trial is typically much larger than the pool of participants that bear its risks, meaning, effectively, that many trials will have tremendous weight on their benefits.

To see the implications of these points, consider, as a toy example, the already‐mentioned proposal to use CHIs with SARS‐CoV‐2 to attempt to accelerate vaccine development, and for simplicity consider mortality as the relevant health benefit and harm. One estimate of the mortality risk of exposing young, healthy people to SARS‐CoV‐2 in the context of a CHI is 0.0025% [22], and one such CHI, eventually conducted in England, required 36 participants [23]. Exposing 36 people to a 0.0025% chance of death yields an expectation of 0.0009 deaths. By contrast, the WHO has estimated that, in the first 2 years of COVID, there were fifteen million excess deaths worldwide [24]. From these numbers, it follows that a SARS‐CoV‐2 CHI conducted early in the pandemic could have achieved a risk‐benefit ratio less than one if it had so much as a one‐in‐three‐hundred‐million chance of reducing total excess deaths of the first 2 years of the pandemic by as little as two percent.^10^ For reference, one in three hundred million is the current chance of winning the Powerball Lottery [25]. So, while most trials fail, and while skeptics were correct that these proposed trials faced significant challenges in completion and translation, it is unclear whether they meant to claim that even success *that* modest was *that* unlikely. But, per the ratio conception outlined above, this is how unlikely even such modest success would need to be for such a trial to fail to attain proportionality.

One might resist this conclusion by insisting that, earlier arguments notwithstanding, the ratio must be much lower than one. But that matters less than it may seem. Imagine insisting that the ratio must be 0.02 or lower, representing the (extremely morally implausible) claim that risks must be justified by benefits fifty times over. Requiring this is equivalent to introducing a fixed constant to the ratio and can correspondingly be cancelled out by equivalent modification of the other terms. In this case, a fifty‐times‐over favorable ratio could be achieved by a SARS‐CoV‐2 CHI with a one in 30 million chance of reducing excess deaths in the first 2 years of the pandemic by 10% (the earlier chance of success has been multiplied by ten, and the magnitude by five, so benefit overall by a factor of fifty). While this is a more demanding bar, it still allows trials with extremely low chances of at best partial success.

SARS‐CoV‐2 CHIs are an admittedly extreme example, as both individual risk and the number of participants required were very small, and being compared against the enormous expected value of even tiny chances to mitigate an ongoing global pandemic. It is nonetheless a fair example, given that the proportionality of those studies was controversial and that controversy motivates the need for further theoretical development. But in terms of broader lessons, it is reasonable to wonder to what extent this will generalize. This case on its own does not show sub‐lottery level chances of success are acceptable in *all* trials. However, there is a great deal of room for chances to be very small without being lottery‐small. For example, a one in three million chance of success remains extremely small, but when plugged into the above would support a balance that is otherwise a hundred times worse.

Furthermore, insofar as the numerical imbalance between participants and potential beneficiaries is a major structural factor driver of these findings, its effect may become more powerful over time. Population change does not directly affect the number of people required for a trial, nor the risks of the interventions administered to them, but does change the number of people who stand to benefit, thereby enhancing the risk‐benefit ratios of trials even in absence of other changes in the underlying social and medical facts. Consider the 50‐fold difference between requiring a ratio of 1 and of 0.02: this is also roughly the factor by which the world population has grown between 0 CE and the present day [26]. While we cannot know the future, if we imagine one containing trillions of humans scattered across the stars, it may be that trials with even the tiniest chances of success could have highly favorable risk‐benefit ratios, at least insofar as they are understood as literally being ratios of magnitudes arrived at by summation.^11^

Having made the basic case for a literal ratio understanding and illustrated its tendency to find trials with large numbers of potential beneficiaries proportionate, I now go on to consider objections and alternatives.

## 7 Objection: Is This Unacceptably Utilitarian?

Some might object that adding up risks and benefits in the above way is objectionably utilitarian. But we need not be utilitarians to accept any of this. As initially emphasized, proportionality is but one requirement among the many that constitute standard research ethics. For example, as I have been at pains to emphasize, this understanding of proportionality is consistent with maintaining that a favorable risk‐benefit ratio *also* includes a separate (absolute, deontic) requirement that individual net risks not be too high. Utilitarianism is distinguished by its denial that anything other than magnitudes of benefit and harm is ever morally relevant. A theory can remain fully non‐utilitarian while agreeing that to answer at least some parts of some questions, magnitudes of benefit and harm should be added up and compared.

## 8 Objection: Is Anything Disproportionate?

A second objection is that the above account trivializes proportionality—if proportionality is consistent with lottery‐level chances of success, what could possibly be disproportionate?

As already noted, not every case will be as extreme as that of SARS‐CoV‐2 CHIs. Not all research aims at ameliorating serious health problems affecting large populations. Some rare diseases research, even if successful, may directly benefit only a handful of people.^12^ Implementation science within the context of a specific population or health system may be beneficial and important but also only relevant to a discrete and relatively small population. Finally, even with respect to trials targeting serious problems affecting worldwide populations, some may genuinely have sub‐lottery level chances of success.

The natural question, then, is where the line will end up falling. Granting that this could only ever be demonstrated to full satisfaction through demanding and thorough empirical work, I nonetheless suggest what seems to me the most plausible conclusion from these preliminary observations, namely, that internal proportionality, while not vacuous, will turn out to be generally easy to attain when it comes to scientifically valid research on widespread health problems. This seems the most likely result just by virtue of the weight of extremely large numbers of potential beneficiaries. And, given that much research is scientifically valid and does address widespread health problems, this reasoning then suggests that the requirement of internal proportionality should only in rare cases be a focus of research ethics review.

Is this an unacceptable result? No. We should not reject an understanding of internal proportionality solely because it implies that, under current conditions, and for much research, the standard is easily met. Under some conditions, it *should* be easily met. Suppose that Novel Alternative Methods, such as the use of computer simulations and more complex artificial biological systems (e.g., “organ on a chip”) advance tremendously within the next decades [27]. These technologies have the potential to significantly improve preclinical safety and efficacy data. If they do, the risk‐benefit ratio of much research would thereby improve. How should we then react? Not by making risk‐benefit ratios much more demanding, to ensure that a significant number of trials would still fail to attain one. Instead, we should say that, given this new reality, nearly all trials would be internally proportionate.

But given that there is nothing conceptually confused about the idea of eventually coming to circumstances under which nearly all human subjects research would be internally proportionate, why think those circumstances don't already obtain? Since the Belmont Report, the world population has roughly doubled; so too have the number of potential research beneficiaries [26]. If the standard is *not* easy to meet now, does that mean it was nearly impossible to meet then? This is the kind of question for a theory of proportionality to decide; without already having such a theory, it is unclear how easy meeting this standard is supposed to be.

Thus, rather than taking it as a ground for rejection, I instead endorse the implication that when it comes to scientifically valid research targeting widespread health problems, the requirement of internal proportionality is generally easy to meet.

Are there viable alternatives?

## 9 Alternative: Brute Intuitions?

When attempting to define proportionality quantitatively, it is difficult to escape the fact of very large numbers of potential beneficiaries. Hence, a natural alternative for those who find the above disturbing is to reject both definition and quantification entirely: perhaps the language of “ratios” and “weighing” gestures at the idea that risks and benefits must both be considered but gives no instructions on how to do so.^13^

The strongest intuitionist stance would be to treat proportionality entirely as a black box: there is nothing to do other than find reasonable, morally sensitive people, give them relevant information, and ask them to make a judgment. But this approach has downsides. Reliance on brute intuition renders it impossible to explain why one intuitive judgment, and not another, is correct, correspondingly difficult to guarantee that any two judges are judging matters in the same way, and impossible to demonstrate when a decision is being made for the right reasons as opposed to invidious, corrupt, or clearly irrelevant ones.

What's worse, we have reasons to think that our intuitions should be unreliable here. Even those who reject summation as an appropriate way of relating individual outcomes to overall ones will generally agree that how many individuals are affected matters, and, similarly, even those who reject expected value as appropriately taking uncertainty into account will agree that how likely a given effect is matters. But if these factors are supposed to play an (imprecise but significant) role in our judgments, then that requires we engage in accurate intuitive reasoning about very large populations and very small chances. For an individual in an ordinary, one‐off decision context the difference between a one in ten thousand chance and a one in ten trillion chance can be effectively ignored; they both might as well be zero. Yet if we are assessing, say, gain‐of‐function research that might cause a global pandemic, that difference is important. Is it credible that our intuitions reliably track these differences? While research ethicists may acquire a greater intuitive facility with probabilities than the general public, expecting this skill to be robust down to hundredths of a percent is optimistic.

## 10 Alternative: Intuitive Comparisons?

The choice between either trying to define and quantify proportionality, on the one hand, or relying on brute, opaque intuitions may seem false. Indeed, the most prominent method for assessing the related‐but‐distinct question of where to locate upper limits on individual net risk is arguably something in between. That method, the method of comparisons, works as follows: one must first find another comparator activity wherein people assume risk, which is both intuitively ethically acceptable and tightly parallel to the research participation in question; one is then entitled to infer that research as risky as that comparator, and no riskier, is permissible. This method does appeal to intuitions, but in a structured way. And given the popularity of this method for assessing upper risk limits, it would be remiss not to address whether an analogue could be used to assess proportionality as well. Indeed, one might hold out hope that such an alternative would allow us to avoid having to quantify amounts of benefit in ways that, perhaps unescapably, will drive results like the above. Here, I argue to the contrary that, whatever its legitimate uses, the method of comparisons has significant limitations and is not promising as a route for assessing proportionality.

First, consider existing attempts to draw comparisons in order to illuminate upper risk limits: London states that legitimate comparators for research must be ones in which people do not enjoy risk (e.g., no extreme sports), where they take on burdens for the benefit of others, and where the people undergoing risk do not understand or control those risks; he suggests looking to public service professions, like volunteer firefighting, but is unsure whether this parallel is close enough [13, pp. 2881–2883]. Miller and Joffe suggest live organ donation as a comparator, as it involves submitting to medical treatment and assuming some risks for the benefit of another—but ultimately conclude that this parallel fails because the connection between participants personal sacrifice and a concrete benefit is significantly more uncertain than it is for donors [28]. Shah et al., in assessing a potential Zika Challenge Trial, elected to compare that research to the most similar thing they could find, other phase 1 research using healthy volunteers (and one thing their survey reveals is that phase 1 research with healthy volunteers is in fact extremely safe, and hence it would be difficult to justify any significant risks in that manner) [29, pp. 10–11].

In drawing such comparisons, we face choices. We could compare against an activity similar to research in any respects that *might be* morally relevant, or just in all respects that *are* morally relevant. Similarly, we could compare against an activity that is *obviously* morally permissible, or just one that *is* (non‐obviously) morally permissible.

Insisting on similarity in all dimensions that might be morally relevant has its advantages. If research is exactly like some other permissible activity in all conceivably relevant respects, then we don't have to know which of those respects really are relevant, and, when coupled with the choice of a comparator that is obviously morally permissible, this makes a strong, theory‐neutral argument for permissibility. But the problem is that while having a nearly exactly similar and obviously permissible comparator is a strong argument for permissibility, the absence of one is not a strong argument against permissibility.

To illustrate, imagine activity A is risky but permissible. It is surely possible that there is no comparator almost exactly like A, simply because the only thing that will ever be exactly like A is A itself. Yet despite the lack of such a comparator, A is, by description, permissible. For instance, I take it that attempting the natural birth of twins is permissible, and that this can be true even if we cannot identify any other equally‐or‐more risky activity which is almost exactly like it. It is plausible that we will be unable to identify such a comparator just because there is nothing out there which is almost exactly like attempting the natural birth of twins.

Or suppose that A does have some comparator, B, which has some risks x and is *obviously* permissible. But suppose also that B *could have* had risks y such that y > x and it still would have been permissible. Looking to the actual risks of B, which are x, and concluding that this is the maximal permissible risk, will lead one to falsely conclude that activities with risk y are impermissible. For instance, recreational pedal cycling is an obviously permissible activity for competent adults to engage in that carries an annual injury rate of ~1.1 per 1,000 cyclists. But it's not as if pedal cycling would have been impermissible if the annual rate of injury had instead been slightly higher at ~1.3 per 1000—which is the actual rate for basketball [30], [table 3, p. 9]. Importantly, this point would remain true even if basketball did not exist to illustrate it, and instead the riskiest actually existing sporting activity we could document was pedal cycling. Even if that were so, inferring that it represented a maximum limit of permissible risk would be a significant moral mistake.

Requiring that research have a near‐exactly similar, obviously permissible comparator is a highly conservative procedure, rarely issuing in false judgments of permissibility but prone to false judgments of impermissibility. This would be unproblematic if it were important to avoid permitting inappropriately risky research and not important to avoid forbidding acceptably risky research. However, forbidding acceptable research that could have improved human health and wellbeing leads to ethically gratuitous morbidity and death.

The conservatism of the comparator method is substantially mitigated when similarity is required only in those dimensions that *are* morally relevant, not in every dimension that *might be*, and when the activities being compared are very near the limit of permissible risk—activities which *are* permissible, although perhaps not *obviously* so. The most charitable interpretation of existing uses of the method may be as aiming to do just that. But, if so, the problem becomes that these comparisons now start from substantive judgments that are inherently tendentious.

In the above examples, I doubt that any one of volunteer firefighting, kidney donation, or phase 1 research with healthy volunteers really are nearly‐impermissibly risky—even if rates of hospital‐borne infection rose slightly tomorrow, thereby making kidney donation slightly riskier, kidney donation would not then become morally impermissible. But, as noted above, if these are activities that could have been riskier while still remaining permissible, adopting their actual risks as an upper limit falsely classifies permissible research as impermissible. And second, I doubt the true relevance of all the dimensions of similarity being required. For instance, as mentioned, London excludes extreme sports because people enjoy playing them, unlike research participation. This choice makes sense insofar as enjoyment of the activity may alter the net risk profile for the individual; however, combining the view that there are upper limits on the ethically permissible assumption of risk with the view that fun cancels risk has its own extremely counterintuitive implications, for example, that there are cases where it would be ethically permissible to base jump off a cliff for fun, but ethically impermissible to base jump off the same cliff to provide lifesaving care to a person who is stranded at the bottom.

Do these criticisms amount to an argument against the method of comparisons itself? Perhaps such difficulties simply call for better and more thoroughly argued choices of comparator. Doing that well is difficult—the task of finding *actually* (but non‐obviously) permissible comparator activities, which are similar not in all dimensions but just in those that are morally relevant, is difficult. Still, nothing here establishes that it is impossible, and, at least for present purposes, I allow that when it comes to determining individual net risk limits the use of such comparisons may be the only game in town. These limits, as some proponents acknowledge, lack a clear theoretical basis.^14^ Therefore, we cannot deduce from theory where to locate them. Yet many are convinced that they are nonetheless real, so it may be that trying to discern their location through a series of intuitive comparisons is the best we can do.

However, with respect to proportionality we are not quite so theoretically at sea. Proportionality involves morally comparing harms and benefits to different groups. While these comparisons are difficult for both empirical and conceptual reasons, they are not impossible and are routinely done in assessing the desirability of policy and social arrangements. Above, I attempted to interpret proportionality in such a way. But assessing the size and distribution of benefits and burdens and then determining whether they are on‐balance desirable, requires, rather than avoids, at least rough quantification, and in carrying it out we will re‐experience, rather than avoid, the facts already noted about the relatively small number of participants required for trials as against the enormous population that stands to benefit.

## 11 Conclusion

This paper has attempted to show that the requirement for proportionality between risk and benefit is currently undertheorized, to disambiguate two kinds of proportionality (internal and external), to outline one promising way of understanding internal proportionality (in terms of a literal ratio of expected health losses and gains), and to show that this understanding has the tendency to render scientifically valid trials targeting widespread health conditions internally proportional even when they are very unlikely to succeed. I have argued that we should accept this implication. I have also argued, relatedly, that the prospects for alternative, intuition‐driven methods of assessing proportionality are bleak.

## Conflicts of Interest

The author declares no conflicts of interest.

## Endnotes

1 Versions of this claim are found in a variety of guidance documents and regulations. In the United States’ code of federal regulations, this is operationalized through the requirement that Institutional Review Boards (IRBs) only approve research when “[the r]isks to subjects are reasonable in relation to [the] anticipated benefits, if any, to subjects, and the importance of the knowledge that may reasonably be expected to result” [1], [§ 46.111(2)]. This faithfully reflects the material on assessment of risks and benefits presented in the Belmont Report [2].

2 Shah et. al. held that “Recognizing the uncertainties, risks from SARS‐CoV‐2 CHIs appear comparable to the risks from some other research and activities similar to research… They also seem to fall below the upper risk limits proposed for research,” [8, p. 832] yet nonetheless reported disagreement “on whether the social value of such CHIs is sufficient to justify the risks at present” [8, p.834]. At the same time, in a later response to Eyal, they separately endorse both proportionality and net risk concerns [9].

3 Notably, the components of a favorable‐risk benefit ratio apply not just to studies considered as a whole package, but to each intervention considered singly. But this complication will not be relevant to the arguments to follow and is hereafter suppressed.

4 Note that, in contrast to the Net Risks view, some maintain that permissible research must be in “equipoise,” a more restrictive condition where, in some specified respect, all interventions a participant might receive are equally good as the best existing clinical care in expectation; Weijer's Component Analysis [12] and London's Integrative Approach [13] are prominent examples. This might be thought to conflict with the text's approach of treating proportionality as a question about the justification of (net) risks. However, these views both allow that there are at least some legitimate impositions of pure research risk, either in the context of entire trials with some risks and no prospect of direct benefit to participants (e.g., phase 1 healthy volunteer research), or when trials include specific interventions with no prospect of direct benefit to participants (e.g., a lumbar puncture to measure pharmacokinetics purely for research purposes after an intervention is administered). Of such pure research risks, they say that the relevant requirements are just the ones already mentioned: that risks imposed purely for research purposes must be both adequately low and justified by the social value the research gains from their inclusion. Hence, they are also committed to making proportionality judgments relating at least some participant risks to social value.

5 Many are parallel to debates in health‐related priority‐setting, for example, over QALY and DALY measures. For instance, we might choose to take an expansive view and count all benefits from a trial as part of its social value, or just health benefits. When adding, we might over‐weigh benefits that accrue to the especially badly off or to those whose rare diseases would not otherwise be cost‐effective to treat. We might employ a discount rate to benefits that occur farther into the future. What follows does not substantially depend on such choices, which is why I do not discuss them further.

6 So, while for example, pediatric studies may include interventions which pose a minor increase over minimal risk, assessing the proportionality of such risks would require separate arguments.

7 Thanks to an anonymous reviewer for raising this objection.

8 I take a group soft paternalist approach to participant autonomy, following Miller and Wertheimer; this approach endorses soft over hard paternalism, but also acknowledges that, in designing feasible social policy, facilitating the autonomy of some will need to be traded off against the restraint of others [16].

9 Some effective altruists writing online have considered this possibility [20]. Whether kidney donation is actually inefficient is not important to this argument; the point is that it could be, and if it were kidney donation would not thereby become unintelligible.

10 15,000,000 * 2% * (1/300,000,000) = 0.001 > 0.0009.

11 Contrariwise, if gradual depopulation begins in mid‐future as current models predict, proportionality will become harder rather than easier to attain. Making it more difficult to conduct beneficial medical research is likely not the most pressing of all concerns with depopulation, but, on this view, it would be among the effects.

12 As one reviewer pressed, rare disease research can develop important knowledge of mechanisms with relevance far beyond the disease itself. This point is well‐taken and such effects are another factor that, if properly accounted for, may make even more research internally proportional. Still, this need not always be true. A study optimizing regimens for symptom relief, for instance, may be unlikely to significantly advance basic science.

13 Emanuel, Wendler, and Grady, in their classic synthesis, state that it is “obvious” that the relevant notion of proportionality is nonquantifiable, though—contra the brute intuition view about to be discussed—they nonetheless enjoin rigor and systematicity [10, p. 2705]. They cite the Belmont Report, which states that the language of balancing and ratios is of a “metaphorical character” [2], [part C, Section 2, The Systematic Assessment of Risks and Benefits].

14 Rid and Wendler, for instance, state that “exactly what justifies and what defines these upper risk limits remains an open question,” [11, p. 165].

## References

[1] “Part 46—Protection of Human Subjects,” US Code of Federal Regulations, https://www.ecfr.gov/current/title-45/subtitle-A/subchapter-A/part-46. 11660819

[2] National Commission for the Protection of Human Subjects of Biomedical and Behavioral Research, *The Belmont Report*, U.S. Department of Health and Human Services, https://www.hhs.gov/ohrp/regulations-and-policy/belmont-report/read-the-belmont-report/index.html.

[3] N. Eyal , M. Lipsitch , and P. G. Smith , “Human Challenge Studies to Accelerate Coronavirus Vaccine Licensure,” Journal of Infectious Diseases 221, no. 11 (June 2020): 1752–1756, 10.1093/infdis/jiaa152. 32232474 PMC7184325

[4] S. A. Plotkin and A. Caplan , “Extraordinary Diseases Require Extraordinary Solutions,” Vaccine 38, no. 24 (May 2020): 3987–3988, 10.1016/j.vaccine.2020.04.039.10.1093/infdis/jiaa152. 32331807 PMC7167540

[5] L. Dawson , J. Earl , and J. Livezey , “Severe Acute Respiratory Syndrome Coronavirus 2 Human Challenge Trials: Too Risky, Too Soon,” Journal of Infectious Diseases 222, no. 3 (July 2020): 514–516, 10.1093/infdis/jiaa314. 32496536 PMC7313940

[6] J. P. Kahn , L. M. Henry , A. C. Mastroianni , W. H. Chen , and R. Macklin , “For Now, It's Unethical to Use Human Challenge Studies for SARS‐CoV‐2 Vaccine Development,” Proceedings of the National Academy of Sciences 117, no. 46 (November 2020): 28538–28542, 10.1073/pnas.2021189117. PMC7682548 33122444

[7] D. P. Sulmasy , “Are SARS‐CoV‐2 Human Challenge Trials Ethical?,” JAMA Internal Medicine 181, no. 8 (August 2021): 1031–1032, 10.1001/jamainternmed.2021.2614. 34096982

[8] S. K. Shah , F. G. Miller , T. C. Darton , et al., “Ethics of Controlled Human Infection to Address COVID‐19,” Science 368, no. 6493 (May 2020): 832–834, at p.832, 834, 10.1126/science.abc1076. 32381590

[9] S. K. Shah , F. G. Miller , T. C. Darton , et al., “Unnecessary Hesitancy on Human Vaccine Tests—Response,” Science 369, no. 6500 (July 2020): 151, 10.1126/science.abc9380. 32646992

[10] E. J. Emanuel , “What Makes Clinical Research Ethical?,” Journal of the American Medical Association 283, no. 20 (2000): 2701–2711, 10.1001/jama.283.20.2701. 10819955

[11] A. Rid and D. Wendler , “A Framework for Risk‐Benefit Evaluations in Biomedical Research,” Kennedy Institute of Ethics Journal 21, no. 2 (June 2011): 141–179, 10.1353/ken.2011.0007. 21696094

[12] C. Weijer and P. B. Miller , “When Are Research Risks Reasonable in Relation to Anticipated Benefits?,” Nature Medicine 10, no. 6 (June 2004): 570–573, 10.1038/nm0604-570. 15170195

[13] A. J. London , “Reasonable Risks in Clinical Research: A Critique and a Proposal for the Integrative Approach,” Statistics in Medicine 25, no. 17 (June 2006): 2869–2885, 10.1002/sim.2634. 16810711

[14] L. Pierson and J. Millum , “Health Research Priority Setting: The Duties of Individual Funders,” American Journal of Bioethics 18, no. 11 (November 2018): 6–17, at p. 7, 10.1080/15265161.2018.1523490. 30475176

[15] R. Y. Chappell , “Pandemic Ethics and Status Quo Risk,” Public Health Ethics 15, no. 1 (April 2022): 64–73, 10.1093/phe/phab031.

[16] F. G. Miller and A. Wertheimer , “Facing Up to Paternalism in Research Ethics,” Hastings Center Report 37, no. 3 (May/June 2007): 24–34, 10.1353/hcr.2007.0044. 17649900

[17] A. A. Marsh , M. Magalhaes , M. Peeler , et al., “Characterizing Altruistic Motivation in Potential Volunteers for SARS‐CoV‐2 Challenge Trials,” PLoS One 17, no. 11 (November 2022): e0275823, 10.1371/journal.pone.0275823. 36322529 PMC9629635

[18] J. Earl , L. Dawson , and A. Rid , “The Social Value Misconception in Clinical Research,” American Journal of Bioethics 25, no. 8 (July 2025): 61–77, 10.1080/15265161.2024.2371119. 39007856

[19] W. H. Lim , K. E. Chan , C. H. Ng , et al., “A Qualitative Systematic Review of Anonymous/Unspecified Living Kidney and Liver Donors' Perspectives,” PLoS One 17, no. 12 (December 2022): e0277792, 10.1371/journal.pone.0277792. 36584032 PMC9803135

[20] J. Kaufman , “Atruistic Kidney Donation,” accessed August 31, 2025, https://www.jefftk.com/p/altruistic-kidney-donation.

[21] A. Rid and M. Roestenberg , “Judging the Social Value of Controlled Human Infection Studies,” Bioethics 34, no. 8 (August 2020): 749–763, 10.1111/bioe.12794. 32844460

[22] D. Manheim , W. Wiȩcek , V. Schmit , and J. Morrison , 1Day Sooner Research Team , “Exploring Risks of Human Challenge Trials for COVID‐19,” Risk Analysis 41, no. 5 (May 2021): 710–720, 10.1111/risa.13726. 33942351 PMC8207107

[23] B. Killingley , A. J. Mann , M. Kalinova , et al., “Safety, Tolerability and Viral Kinetics During SARS‐CoV‐2 Human Challenge in Young Adults,” Nature Medicine 28, no. 5 (May 2022): 1031–1041, 10.1038/s41591-022-01780-9. 35361992

[24] “14.9 Million Excess Deaths Associated With the COVID‐19 Pandemic in 2020 and 2021,” World Health Organization, May 5, 2022, https://www.who.int/news/item/05-05-2022-14.9-million-excess-deaths-were-associated-with-the-covid-19-pandemic-in-2020-and-2021.

[25] “How Hard Is It to Win The Lottery? Odds to Keep in Mind as Powerball and Mega Millions Jackpots Soar.” *AP News*, July 19, 2023, https://apnews.com/article/powerball-mega-millions-winning-odds-numbers-a3e5a8e8e7ed15d7500c1d6acdab6785.

[26] M. Roser and H. Ritchie , “World Population Over Time, Our World in Data,” last modified June 1, 2023, https://ourworldindata.org/population-growth-over-time.

[27] M. Bertagnolli , “Statement on Catalyzing the Development of Novel Alternative Methods,” NIH, last reviewed December 10, 2024, https://www.nih.gov/about-nih/who-we-are/nih-director/statements/statement-catalyzing-development-novel-alternatives-methods.

[28] F. G. Miller and S. Joffe , “Limits to Research Risks,” Journal of Medical Ethics 35, no. 7 (July 2009): 445–449, 10.1136/jme.2008.026062. 19567696

[29] S. K. Shah , J. Kimmelman , A. Drapkin , et al., “Ethical Considerations for Zika Virus Human Challenge Trials: Report and Recommendations,” February 2017, https://www.niaid.nih.gov/sites/default/files/EthicsZikaHumanChallengeStudiesReport2017.pdf.

[30] Y. Sheu and H. Hedegaard , “Sports‐ and Recreation‐Related Injury Episodes in the United States, 2011–2014.” *National Health Statistics Reports*, 99 (November 2016), https://www.cdc.gov/nchs/data/nhsr/nhsr099.pdf. 27906643

